# A ZnO/porous GaN heterojunction and its application as a humidity sensor†

**DOI:** 10.1039/c8na00243f

**Published:** 2018-12-21

**Authors:** Chao Wang, Hui Huang, Miao-Rong Zhang, Wei-Xing Song, Long Zhang, Rui Xi, Lu-Jia Wang, Ge-Bo Pan

**Affiliations:** Division of Interdisciplinary Research, Suzhou Institute of Nano-Tech and Nano-Bionics, Chinese Academy of Sciences 215123 Suzhou P. R. China gbpan2008@sinano.ac.cn; School of Nano Technology and Nano Bionics, University of Science and Technology of China 230026 Hefei P. R. China; Department of Chemistry, Capital Normal University 100048 Beijing P. R. China songwx@cnu.edu.cn

## Abstract

A heterojunction of ZnO/porous GaN (ZnO/PGAN) was fabricated and directly applied to a diode-type humidity sensor. ZnO disks were loaded onto PGAN using a spraying process. The structure and surface morphology of the ZnO/PGAN were characterized using X-ray diffraction and scanning electron microscopy. The heterojunction displayed an excellent diode nature, which was investigated using photoluminescence spectra and *I*–*V* characteristics. The excellent transport capability of ZnO/PGAN contributes to enhanced electron transfer, and hence results in high sensitivity and quick response/recovery properties under different relative humidity (RH) levels. In the range of 12–96% RH, a fast sensing response time as low as 7 s and a recovery time of 13 s can be achieved. The simple design of a ZnO/PGAN based humidity sensor highlights its potential in various applications.

## Abstract 2

A new humidity sensor based on a heterojunction of ZnO/PGAN is fabricated by a simple spraying process and the heterojunction can be directly applied to a diode-type humidity sensor.

## Graphical abstract

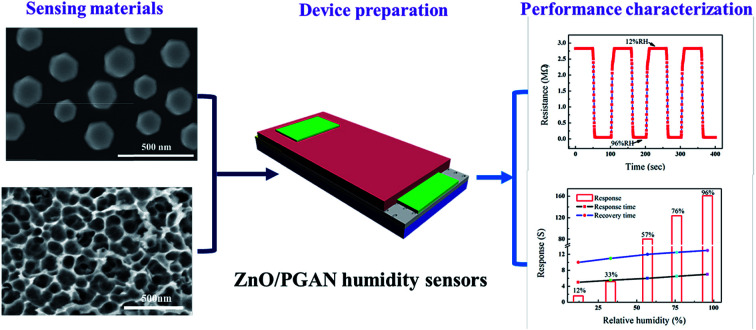

## 1 Introduction

Humidity sensors (HSs) for detecting relative humidity (RH) have attracted more and more attention because of their extensive practical and potential application in industrial process control, medicine and food production, human health and environmental protection, and so on.^1–4^ In the past few years, semiconducting metal oxides have been considered promising candidates for gas-sensing applications because of their high sensitivity, easy fabrication methods and low cost.^5^ Nanostructured zinc oxide (ZnO) has drawn significant attention due to its vast applications in solar cells, gas sensors, field emission devices, humidity devices and photovoltaic devices.^6,7^ On the other hand, nanostructured ZnO has the advantages of abundant surface morphology, good chemical and thermal stability and superior electrical properties.^8,9^ All these features of ZnO are very useful for constructing composite nanosystems with enhanced humidity-sensing properties.^10^ Alumina (Al_2_O_3_) is generally used as a substrate to fabricate sensing devices as it is a good insulator and is chemically inert.^11,12^ Recently, ZnO was successfully formed on porous Si (PSI).^13^ However, the instability of PSI in harsh and corrosive environments leads to the limitation of the application of humidity sensors. Porous GaN (PGAN) can be used as an alternative material for humidity sensors. The interest in this material is mainly due to its high surface to volume ratio, super chemical stability and wide direct bandgap.^14^ More importantly, PGAN serving as a substrate^15^ not only supports the sensing materials, but also improves sensing performance because of its high electron mobility.

In recent years, there have been quite a few explorations on a single humidity-sensing material based on ZnO nanomaterial.^16,17^ The performance of HSs, in terms of sensitivity and reproducibility, has been improved to a certain extent.^18^ With regard to sensing parameters such as sensitivity, reproducibility, response time and recovery time, the majority of the humidity sensors reported to date show excellent performance in a parameter or two; however, they still lack overall performance involving all parameters. Generally, humidity sensors of metal oxide nanostructures have been developed on the basis of electrical resistance detection mode.^9^ The synergistic effect between each component of the heterojunction may compensate for the drawback of a single functional material and present new sensing properties.^19^ Significant efforts have been invested by researchers in enhancing the heterojunction of ZnO, which has shown rapid electron transfer for material films.^20^ PGAN with high surface area has emerged as a potential framework matrix for supporting a variety of metal and metal oxides for their use in humidity sensing applications.^21^ The relatively high specific surface area of PGAN provides more effective electron transfer. The fast electron transfer helps in improving the performances of gas sensing such as response/recovery time and hysteresis of the HS. A ZnO/porous GaN (ZnO/PGAN) heterojunction has been applied to biosensing.^22,23^ However, to the best of our knowledge, such a heterojunction based on PGAN for HSs is rarely reported. We expect that superior humidity-sensing properties can be achieved by combining two semiconductors ZnO and GaN to form heterojunction nanostructures.

Herein, a HS based on a ZnO/PGAN heterojunction as the performance-enhancement sensing layer is demonstrated, which exhibits high response and fast response and recovery time over a wide range of RH levels. Humidity sensors show sensitivity from a low (12% RH) to high (96% RH) humidity range with rise and fall time of 7 s and 13 s, respectively. ZnO/PGAN heterojunction devices are becoming promising candidates for HSs due to their excellent diode nature. A possible charge transport mechanism based on physisorbed and chemisorbed water layers is proposed to explain the measured humidity sensing response at room temperature.

## 2 Experimental

### 2.1 Chemicals

Sodium dodecyl sulfate (SDS), cetyltrimethylammonium bromide (CTAB), sodium hydroxide (NaOH), zinc nitrate hexahydrate (Zn(NO_3_)_2_·6H_2_O) and ethanol (C_2_H_5_OH) were used to prepare nano-zinc oxide, and were analytical reagents (AR). Lithium chloride (LiCl), magnesium chloride (MgCl_2_), sodium bromide (NaBr), sodium chloride (NaCl), and potassium sulfate (K_2_SO_4_) were used to obtain various constant relative humidity levels, and were guaranteed reagents (GR). All chemicals were purchased from Sinopharm Chemical Reagent Co., Ltd and used without any further purification.

### 2.2 Synthesis of ZnO disks

In a general procedure for synthesizing ZnO disks, the same molar amounts of SDS and CTAB and 0.08 g NaOH were dissolved in 20 mL of deionized water with vigorous stirring to obtain a transparent solution. 0.89 g Zn(NO_3_)_2_·6H_2_O was dissolved in 20 mL of deionized water and then added to the above transparent solution. After stirring of 20 min, the solution was transferred into a 50 mL Teflon-lined stainless steel autoclave and maintained at 120 °C for 12 h. After the autoclave was cooled to room temperature, the white precipitate was separated from the solution by centrifugation and washed three times with ethanol and deionized water. The sample was dried at 80 °C and then collected for structure characterization and sensor fabrication.

### 2.3 Materials characterization

The surface morphology and chemical composition of the as-prepared ZnO disks were characterized by scanning electron microscopy (SEM, Hitachi S-4800) and energy dispersive spectroscopy (EDS, Quanta FEG 250), respectively. The crystal structure of the ZnO/PGAN heterojunction was obtained by X-ray diffraction at a scanning rate of 0.1° s^−1^, using Cu Kα radiation (XRD, Bruker D8 Advance powder X-ray diffractometer). Photoluminescence measurements were carried out using a Princeton Instruments SP2500i spectrometer at an excitation wavelength of 325 nm in air at room temperature.

### 2.4 Fabrication of the ZnO/PGAN heterojunction sensing device

**Fig. 1:**
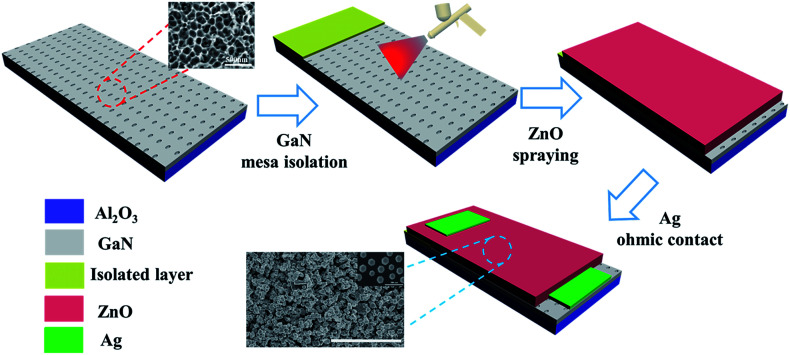
Schematic illustration of the fabrication process of ZnO/PGAN humidity sensors.

The process is schematically illustrated in Fig. 1. An etched GaN film with a 500 nm-thick Al_2_O_3_ layer was used as the substrate. First, a layer of insulating paint film which provides good isolation was deposited by spraying on the PGAN substrate with a mask. Then, a thick micro-structured zinc oxide film was gradually deposited by controlling the spraying speed to balance the spraying and solvent evaporation time. It is important to maintain this balance by properly controlling the airbrush pressure, moving speed of the spray-gun, and the distance between the spray-gun and the substrate. After the ZnO spraying process, the ZnO/PGAN was calcined at 400 °C for 2 h in air atmosphere. In the end, two neighboring Ag electrodes with a width of 0.5 mm were subsequently deposited on the ZnO layer, and were fabricated on the surface of GaN as bottom electrodes, and a piece of Ag foil was placed on top of the ZnO layer as the top electrode.

### 2.5 Sensor measurements

**Fig. 2:**
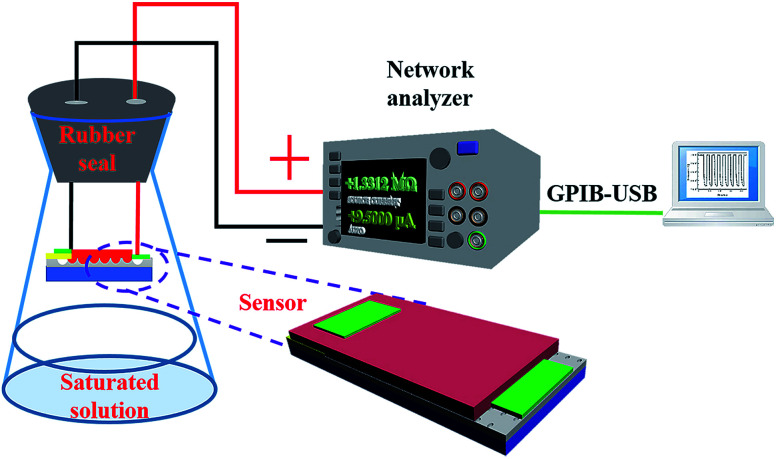
Diagram of the experimental setup for humidity sensing.

All sensing measurements were performed under ambient conditions and room temperature (about 25 °C). A self-developed system with an RH-controllable environment was used for RH sensing measurements, as shown in Fig. 2. The as-prepared HS was placed in the RH-controlled environment and the resistance-time characteristics under a constant bias from a Keithley 2450 voltage source were evaluated. The controlled humidity environment was obtained by using saturated salt solutions of LiCl, MgCl_2_, NaBr, NaCl, and K_2_SO_4_ in rubber-sealed flasks, which yielded approximately 12, 33, 57, 76, and 96% RH at room temperature, respectively.^24^ These RH levels were monitored using a standard hygrometer. For sensing measurements, the sensing device was placed in the rubber-sealed flask and a specific RH% was achieved from saturated salt solutions at room temperature. Two conducting wires with a portion firmly sealed in the rubber seal were connected between the device and an external power supply. The resistance of the device was measured at a bias of 2 V under steady state conditions. The sensing response (*S*) was defined as *S* = (*R*_L_ − *R*_H_)/*R*_H_, where *R*_L_ is the sensor resistance at a comparably lower RH% and *R*_H_ is the sensor resistance at a comparably higher RH%. The transmission measurements of the sensors were investigated at various relative humidity (RH) levels using a network analyzer (2450, Keithley), which was controlled by KickStart software through general purpose interface bus (GPIB).

## 3 Results and discussion

### 3.1 Morphology and structural analysis

**Fig. 3:**
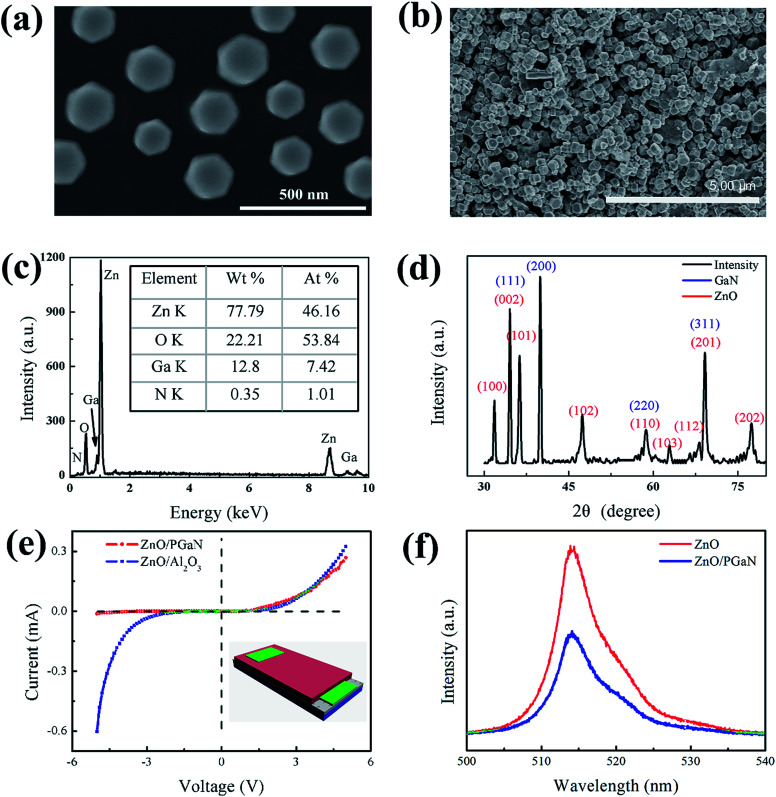
(a) SEM image of ZnO disks on silicon wafer. (b) SEM image of ZnO disks sprayed on PGAN. (c) EDS analysis results for all the area of the material in (b). (d) XRD pattern of ZnO/PGAN. (e) *I*–*V* characteristics of the ZnO/PGAN heterojunction and ZnO/Al_2_O_3_ samples measured at room temperature exposed to atmosphere. (f) Photoluminescence spectra of pristine ZnO and ZnO/PGAN heterojunctions.

The SEM images of the well dispersed ZnO disks and the surface of the ZnO/PGAN device are displayed in Fig. 3(a and b). As shown in Fig. 3(a), the ZnO disks were entirely uniform hexagonal structures, which were obtained *via* a surfactant-assisted method. The diameter of the ZnO disks varies from 150 to 220 nm. Fig. 3(b) clearly shows the disk structure of ZnO on the surface of PGAN. By spraying, the ZnO disks were deposited on the layer of PGAN. The airbrush pressure, speed of the spray-gun and the distance between the spray-gun and the substrate play an effective role in the thickness of the sensitive material.^25^ It is important to maintain this balance by properly controlling the airbrush pressure, moving speed of the spray-gun and spraying distance between the spray-gun and the substrate. As shown in Fig. 3(c), the materials properties of the ZnO/PGAN heterojunction were analyzed by EDS. In addition to the Zn and O signals, Ga and N signals from the GaN substrate were also detected by the EDS analysis, which confirms that the ZnO/PGAN heterojunction has been prepared without other dopants or impurities. In the X-ray diffraction (XRD) pattern (Fig. 2(d)), no impurity peaks belonging to ZnO or GaN were detected. The diffraction peaks at 2*θ* = 34.47°, 40.02°, 57.89° and 69.14° can be ascribed to the (111), (200), (220), (311), (222) facets of GaN, respectively. The diffraction peaks at 2*θ* = 31.77°, 34.42°, 36.25°, 47.54°, 56.60°, 62.86°, 67.96°, 69.09° and 76.95° correspond to the (100), (002), (101), (102), (110), (103), (112), (201) and (202) facets of zincite ZnO (JCPDS no. 36-1451), respectively. Fig. 3(e) shows the typical *I*–*V* characteristics of the ZnO/PGAN heterojunction device. Compared with the *I*–*V* characteristics of a ZnO/Al_2_O_3_ device, obvious rectifying behavior of a diode was observed in the ZnO/PGAN heterojunction device. The ZnO/Al_2_O_3_ device was fabricated on Al_2_O_3_ and two pieces of Ag foil were placed on top of the ZnO disks as top electrodes. The structure of the ZnO/PGAN heterojunction device is shown in the inset of Fig. 3(e), which was fabricated on GaN as bottom electrodes, and a piece of Ag foil was placed on top of the ZnO disks as the top electrode. As shown in Fig. 3(e), the *I*–*V* characteristics of ZnO/Al_2_O_3_ device is the general electrical resistance detection mode.^26,27^ But they are almost the general electrical resistance detection mode. As a diode, its basic characteristics will amplify the electrical signal.^9^ Therefore, designing a diode type humidity sensor using a ZnO/PGAN heterojunction device is very interesting because of its excellent diode characteristics. At the same time, as shown in Fig. S1,† PGAN possesses a large surface area due to the porous nanostructure, which will enhance molecular contact and electronic transport. By measuring the pore size on the SEM image, it was found that the pore size of PGAN is between 50 nm and 225 nm with an average value of 116 nm. The surface area and porosity of PGAN were measured (Fig. S1c†), and the surface area and pore size were 18.21 m^2^ g^−1^ and 118 nm, respectively. In addition, the photoluminescence spectra were recorded and are shown in Fig. 3(f). Compared with ZnO, ZnO/PGAN exhibits strong fluorescence quenching, and the result indicates that the recombination of photoinduced electrons and holes is suppressed as electrons of ZnO are easily extracted by PGAN. PGAN with a unique characteristic of in-plane electrical conductivity is crucial in improving gas sensing performance.^21^ It represents typical charge separation at surface junctions.

### 3.2 Characterization of the ZnO/PGAN heterostructure device

**Fig. 4:**
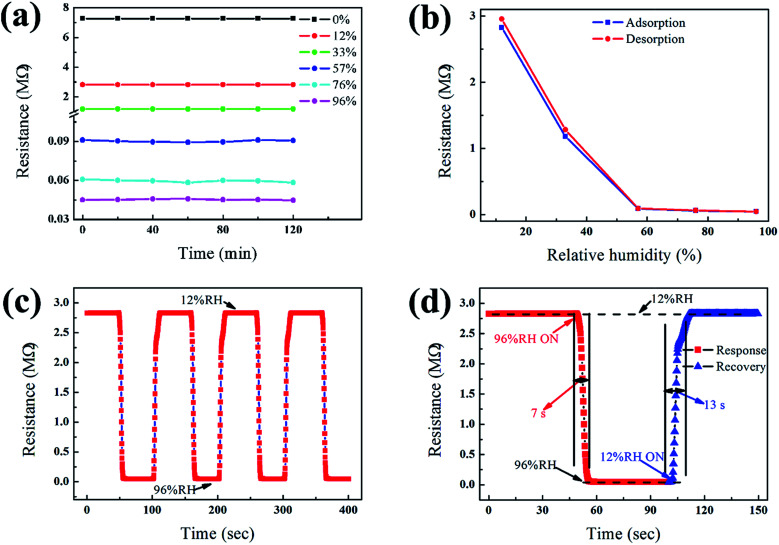
(a) Stability of the measured resistance of the as-prepared sensor with the RH% in the range of 12–96%. (b) Typical hysteresis characteristics of the ZnO/PGAN heterojunction sensor. (c) Response and repeatability characteristics of the sensor when the relative humidity is changed rapidly between 12% RH and 96% RH. (d) Showing the response time and recovery time from 96% RH to 12% RH.

The RH sensitivity of the ZnO/PGAN heterojunction sensor was evaluated. Stability of performance is very important for any type of gas sensor. Here, we investigated the stability of the ZnO/PGAN heterojunction HS under a constant humidity level of 0% RH, 12% RH, 33% RH, 57% RH, 76% RH, 96% RH, respectively. Fig. 4(a) shows the stability of the measured resistance of the as-prepared sensors under different ambient RH levels. These results indicate that resistance variation is less than 5% at each humidity level. The device maintains its stable performance and sensitivity with very little drift and hysteresis for continuous sensing for over 2 h, demonstrating its stability and reliability. Fig. 4(b) shows the hysteresis characteristics in the water adsorption and desorption curves for the ZnO/PGAN sensor. The hysteresis effect is a common phenomenon for metal oxide and nanocomposite-based gas sensors.^24,28^ Minimizing the hysteresis effect is essential for practical sensing applications. In particular, the hysteresis phenomenon is induced by chemisorption on the surface of the sensing element in the initial stage, and it is not easily influenced by exposure to humidity or removed by humidity, which could be just thermally desorbed.^24^ As the RH% decreases, the adsorbed water might not be completely removed, resulting in the hysteresis effect. Hysteresis is defined as the percentage difference in the final settling point of relative humidity when approached from above to when it is approached from below. In the experiment, it was found that the ZnO/PGAN device exhibited an acceptable hysteresis value less than 5%.

Sensors with fast response and recovery are desirable for practical applications. We thus investigated the response speed of the HS subjected to a cyclic change of humidity level. One such example is presented in Fig. 4(c). The sensor shows an extremely excellent repeatability after four cycling tests. The baseline of 96% remains unchanged. The response time is defined as the time to achieve 90% of the total complex impedance changing from 12% to 96% RH and the recovery time is defined from 96% to 12% RH respectively. Fig. 4(d) reveals the details of the rise and fall of resistance of the sensor to humidity change; the sensor exhibits fast response, with a rise time of 7 s when the RH is decreased from 96% to 12%.

Table 1 shows the comparison of the sensitivity and recovery speed of our device with ZnO-based humidity sensors reported in the literature. Compared to other humidity sensors, the ZnO/PGAN based humidity sensor shows improved overall performance involving all parameters. When humidity is switched from 12% RH to 96% RH, a response time of 7 s indicates relatively high sensitivity, which was mainly dominated by the permeation time of moisture to the GaN layer. Our device shows potential for fast humidity sensing in a large RH change range. Most notably, the recovery time is 13 s when humidity is switched from 12% RH to 96% RH. The recovery process of humidity sensors is slow and is mostly dominated by the desorption time of moisture from the ZnO film. However, the recovery time of ZnO/PGAN based humidity sensors is still faster than that of some ZnO based humidity sensors reported in the literature. Considering that the ZnO/PGAN based humidity sensor has only two components, high sensitivity can be mainly ascribed to the enhanced dispersity of ZnO disks and the special PGAN. The characteristic of high electron mobility of GaN can accelerate the electron transfer rate^21^ to make ZnO quickly react with humidity. Therefore, the ZnO/PGAN heterojunction presents potential for fast humidity sensing.

**Table 1:** Performance comparison with previous humidity sensors

Sensor type	Measuring range (%)	Sensitivity	Response time (s)	Recovery time (s)	Reference
Tetrapod-ZnO	5–95	530	36	17	2
ZnO/GO	10–80	15.9 kHz/% RH	<1	19	35
ZnO nanorods	12–97	183	3	20	36
Cd/ZnO	Air–95	—	3	5	37
Al_2_O_3_/ZnO	10–90	—	85	285	38
Al/ZnS	30–90	200	95	209	39
ZnO/GaN	12–96	161	7	13	This work

**Fig. 5:**
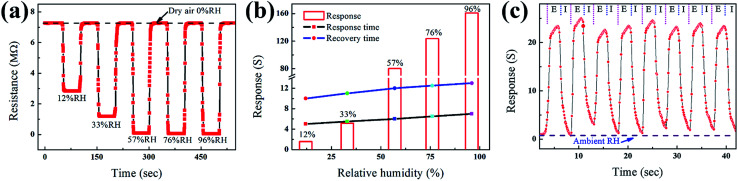
(a) The dynamic humidity sensing behavior of the ZnO/PGAN based device. (b) The sensitivity, response time and recovery time of the ZnO/PGAN HS under different RH. (c) Variations in the exhaled breath of a healthy person (E and I refer to the exhaling and inhaling, respectively).

The HS should also display high sensitivity and fast response/recovery properties under different humidity conditions. Fig. 5 shows the sensitivity and response/recovery time of the as-prepared HS. The dynamic responses of the sensor exposed to different humidity levels are shown in Fig. 5(a). The resistance of the ZnO/PGAN based device decreased along with increasing humidity from 12 to 96% RH. The response is fast and reversible at room temperature. As shown in Fig. 5(b), at 12, 33, 57, 76, and 96% RH, the sensitivities are 1.6, 5.2, 80.4, 123.6 and 161.0, respectively. Meanwhile, at 12% RH, the response and recovery time are 4.5 s and 12 s, respectively. Both response time and recovery time for ZnO/PGAN based sensors slightly increase as the humidity is raised.

Based on the excellent sensing performance, such a humidity sensor based on PGAN has been demonstrated in human breath testing in Fig. 5(c). During exhalation the humidity is expected to be different (usually higher) compared to inhaled (ambient) air. The variations in the RH value are monitored by holding the ZnO/PGAN humidity sensor at a distance of 3.5 cm from the nose while breathing normally. The results displayed that the sensitivity of the device exhibited a sharp rise during exhaling and dropped to ambient value while inhaling, which was nearly repetitive corresponding to the breathing cycles, as shown in Fig. 5(c).

**Fig. 6:**
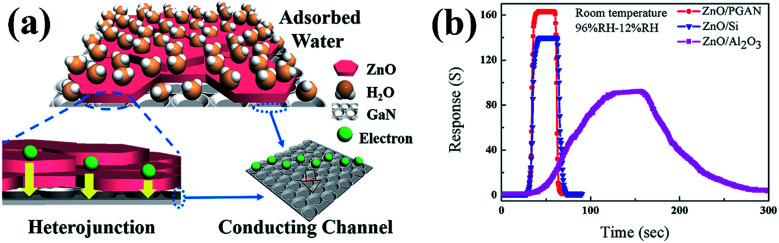
(a) Schematic of H_2_O molecule interaction with the ZnO/PGAN HS. Electron transport on the ZnO/PGAN heterojunction (bottom-left) and effective electron transport on GaN (bottom-right). (b) Response curves of humidity sensors based on ZnO/PGAN, ZnO/Si and ZnO/Al_2_O_3_.

The humidity sensing mechanism is explained on the basis of the conduction mechanism in Fig. 6. According to the literature,^29^ a zinc oxide-based humidity sensor has three sensing states as follows. When the RH is low, the conduction mechanism is mainly electronic conductance. When the RH is at a medium level, the conduction mechanism is mixed electronic–ionic conductance. When the RH becomes high, ionic conductance becomes important. However, the carrier flow of the diode is the basic electronic conductance. Compared with traditional humidity sensors, the difference in the sensing mechanism of the present system is attributed to the structure of the diode, which always exhibits electronic conductance. Therefore, although the sensing mechanism of the zinc oxide sensitive layer changes with humidity, due to the current behavior of the diode, the overall sensing mechanism of the ZnO/PGaN humidity sensor is still electronic conductance.

In the current research, it is noted that the response time and recovery time reflect the ability of the ZnO/PGAN based device to desorb and adsorb water vapor, respectively. Water molecule adsorption and desorption occur at different energy levels. Adsorption is an exothermic process. In contrast, desorption is an endothermic process that requires external energy for water to further depart from the surface of ZnO/PGAN. As show in Fig. 6(a), humidity sensing is related to a moisture-adsorption and desorption process.^30,31^ When exposed to humidified air at room temperature oxygen is adsorbed in the forms of O_2_(ads) and O_2_^−^(ads). During this phase, the impedance of the sensor increases. These oxygen species are then available for reaction with reducing species, like moisture.^16^ The amount of adsorbed water molecules increases with increasing RH. When ZnO/PGAN based sensors are exposed to a humid atmosphere, a large amount of H_2_O molecules are adsorbed on the surface of the ZnO film and PGAN. The porous structure of ZnO/PGAN based sensors enables superior gas sensing performance toward humidity. At low RH values, the large surface area of the first layer of the surface with active OH groups will be filled with water molecules, which can be explained using the Langmuir model.^32^ Due to the lack of sufficient water molecules, a film of adsorbed water cannot be formed on the surface of the ZnO disk. With increasing RH values to a medium level, the first layer of chemisorbed water is formed, and subsequent layers of water molecules are physically adsorbed. The physisorbed water near the chemisorbed water layer can decompose into H_3_O^+^ and OH^−^ ions because of the high electrostatic field in the chemisorbed layer. Therefore, n-doping characteristics can be observed when H_2_O molecules are adsorbed. Because of the n-ZnO film and PGAN, the carrier density can be enhanced by the adsorbed H_2_O molecules. The electrical response depends on the number of water molecules adsorbed on the sensor surface. At high RH values, the adsorption of water molecules on the sensor surface is governed by a multilayer adsorption process. Hence, the device becomes more conductive due to an increased number of free electrons and in-plane electrical conductivity of PGAN.^21^ Because of the diode structure of the ZnO/PGaN heterojunction, the carrier flow of diode is mainly electronic conductance. As previously reported, the reverse current of a heterojunction is very sensitive to the barrier height and barrier width, and thus a small variation in barrier height and width caused by gas adsorption can significantly change the current.^30,33,34^ Under these conditions, the current of the ZnO/PGAN heterojunction will greatly increase when the device is exposed to a humid atmosphere, which leads to a higher sensitivity compared with the value under forward voltage. Compared with ZnO, ZnO/Si and ZnO/Al_2_O_3_, the performance of the sensor based on ZnO/PGAN is obviously better than that of other materials as shown in Fig. 6(b). Therefore, both conduction and electrolysis of the adsorbed water in ZnO/PGAN based humidity sensors improve due to the heterojunction of ZnO/PGAN.

## 4 Conclusions

In summary, we fabricated a ZnO/PGAN based humidity sensor. Both the sensor fabrication and its operation were conducted at room temperature. The sensor exhibited high sensitivity and quick response/recovery properties at room temperature due to its excellent diode characteristics. The ZnO/PGAN based humidity sensor shows high sensitivity at a broad humidity range of 12–96% RH with a response time of 7 s and a recovery time of 13 s. Our results demonstrate that ZnO/PGAN based humidity sensors are suitable for application in various fields such as health and environmental humidity monitoring at room temperature.

## Conflicts of interest

There are no conflicts to declare.

## Supplementary Material

NA-001-C8NA00243F-s001
