# Supplementary material for: A ZnO/porous GaN heterojunction and its application as a humidity sensor
Source: Nanoscale Adv. 2018 Dec 21;1(3):1232–9. doi: 10.1039/c8na00243f (PMC9473300; doi:10.1039/c8na00243f)
Supplement: NA-001-C8NA00243F-s001 [file NA-001-C8NA00243F-s001.pdf]

Electronic Supplementary Material (ESI) for Nanoscale Advances.
This journal is © The Royal Society of Chemistry 2018

Electronic Supplementary Information

ZnO/Porous GaN Heterojunction and its Application as Humidity Sensor

Chao Wang[a,b], Hui Huang[a,b], Miao-Rong Zhang [a], Wei-Xing Song*[c], Long Zhang[a], Rui Xi [a], Lu-Jia Wang [a], Ge-Bo Pan*[a]

[a] Division of Interdisciplinary Research, Suzhou Institute of Nano-Tech and Nano-Bionics, Chinese Academy of Sciences, 215123 Suzhou, P. R. China

[b] School of Nano Technology and Nano Bionics, University of Science and Technology of China, 230026 Hefei, P. R. China

[c] Department of Chemistry, Capital Normal University, 100048 Beijing, P. R. China

* Corresponding authors.

E-mail addresses: songwx@cnu.edu.cn (W.-X. Song), gbpan2008@sinano.ac.cn (G.-B. Pan).

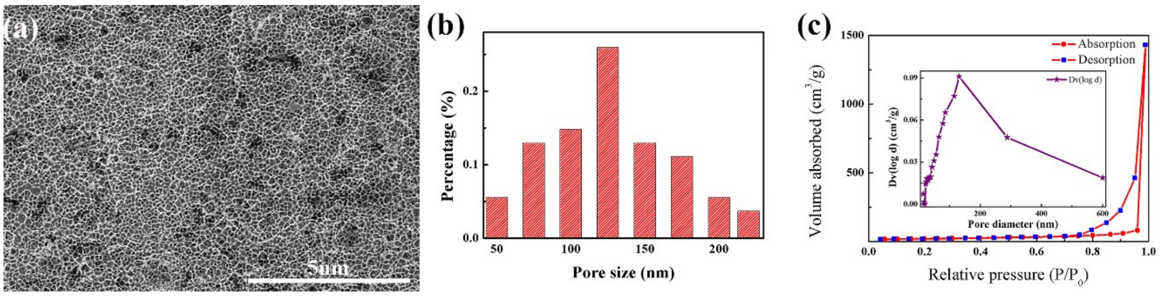

Fig. S1 (a) SEM images of PGAN. (b) Pore size distribution histograms of PGAN. (c) N_2 adsorption-desorption isotherm curve and pore size distribution (inset) of PGAN.
